# Distinct neural correlates of morphosyntactic and thematic comprehension processes in aphasia

**DOI:** 10.1093/braincomms/fcaf093

**Published:** 2025-03-24

**Authors:** Sabrina Beber, Rita Capasso, Chiara Maffei, Marco Tettamanti, Gabriele Miceli

**Affiliations:** Center for Mind/Brain Sciences—CIMeC, University of Trento, Rovereto 38068, Italy; Brain Associates, Roma 00195, Italy; Athinoula A. Martinos Center for Biomedical Imaging, Massachusetts General Hospital, Harvard Medical School, Boston 02129, MA, USA; Department of Psychology, University of Milano-Bicocca, Milano 20126, Italy; Center for Mind/Brain Sciences—CIMeC, University of Trento, Rovereto 38068, Italy; Brain Associates, Roma 00195, Italy

**Keywords:** sentence comprehension, lesion-symptom mapping, morphosyntactic processing, thematic role assignment, thematic re-analysis

## Abstract

Functional neuroimaging studies in neurotypical subjects correlate sentence comprehension to a left fronto-temporo-parietal network. Recent voxel-based lesion-symptom mapping (VLSM) studies of aphasia confirm the link between sentence comprehension and a left posterior region including the angular gyrus, the supra-marginal gyrus and the postero-superior division of the temporal lobe but support left pre-frontal involvement inconsistently. However, these studies focus on thematic role assignment without considering morphosyntactic processes. Hence, available VLSM evidence could provide a partial view of the neurofunctional substrate of sentence comprehension. In the present VLSM study, both morphosyntactic and thematic processes were evaluated systematically and in the same sentence types in each participant, to provide a more detailed picture of the sentence comprehension network. Participants (33 patients with post-stroke aphasia and 90 healthy controls) completed a sentence–picture matching task in which active and passive, declarative reversible sentences were paired with morphosyntactic, thematic and lexical-semantic alternatives. Phonological short-term memory tasks were also administered. Aphasic participants were selected from an initial pool of 70 because they scored below norm on thematic foils (*n* = 18) or on thematic and morphological foils (*n* = 15), but within the norm on lexical-semantic foils. The neurofunctional correlates of morphosyntactic and thematic processes were starkly distinguishable. Pre-frontal areas including the inferior and middle frontal gyrus were involved directly in processing local morphosyntactic features and only indirectly in thematic processes. When these areas were damaged, morphosyntactic errors always co-occurred with thematic errors, probably because morphosyntactic damage disrupts the assignment of grammatical roles and ultimately that of thematic roles. Morphosyntactic errors were not influenced by word order canonicity. In contrast, selective thematic role reversals were linked to temporal and parietal damage and were significantly influenced by word order, occurring on passive more than on active sentences. An area including the angular and supra-marginal gyrus was critical for processing non-canonical word order. In sentence comprehension, pre-frontal regions are critical for processing local morphosyntactic features (at least in simple declarative sentences). Temporal and parietal regions are critical for thematic processes. Postero-superior temporal areas are involved in retrieving verb argument structure. Parietal areas are critical for assigning morphosyntactically analysed constituents to the appropriate thematic role, thus serving a crucial function in thematic re-analysis. Each area plays a prevailing but not exclusive role in these processes, interacting with other areas in the network and possibly providing both the language-specific and the domain-general resources needed at various stages of sentence comprehension.

## Teaser

Beber *et al*. investigate the neurofunctional correlates of sentence comprehension in individuals with aphasia. Results link pre-frontal regions to local morphosyntactic processes (e.g. noun–verb agreement) and temporo-parietal regions to establishing *who does what to whom*, with parietal regions being critical for processing sentences with non-canonical word order (e.g. passives).

## Graphical Abstract

**Graphical Abstract:**
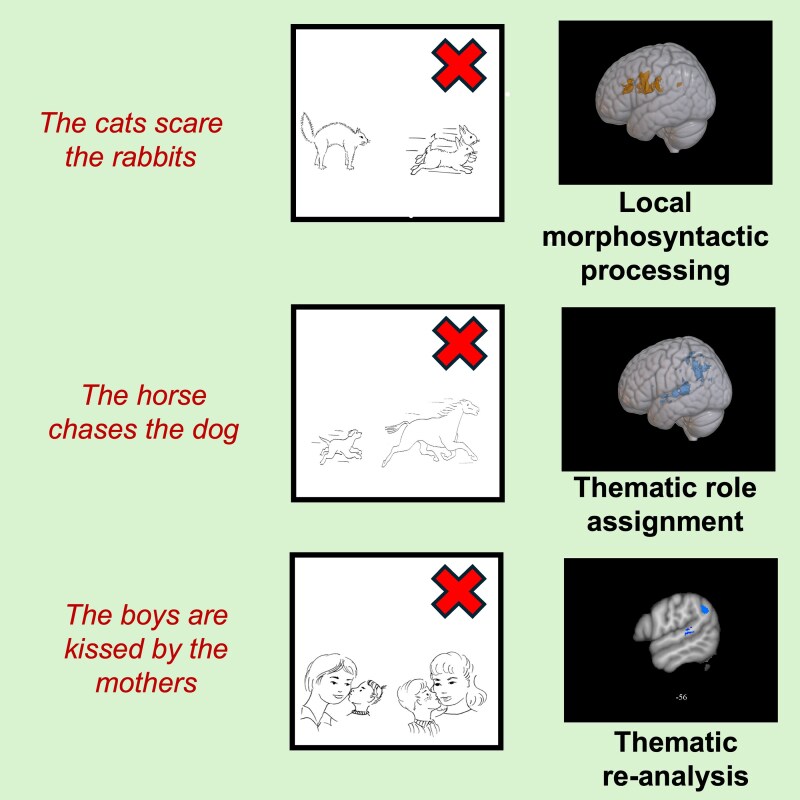

## Introduction

Uncovering the mechanisms that underlie natural human language processes based on evidence from aphasia is a daunting challenge. This is especially true for studies of sentence processing that investigate complex linguistic knowledge and must consider how it integrates with non-linguistic skills. Investigations have focused on semantically reversible stimuli, as in these sentences more than one argument can be the doer or the receiver of the action, and therefore syntactic mechanisms are necessary to assign the thematic roles of agent and theme. For example, in *The boy hugs the girl,* syntax assigns the agent role to *boy* and the theme role to *girl*, even though in real life roles could be reversed. In active sentences, canonical word order facilitates comprehension, as the first noun is both the grammatical subject and the thematic agent.^1^ When the same event is expressed in the passive voice, as in *The girl is hugged by the boy*, however, word order is non-canonical, because the first noun is the grammatical subject but takes the theme, not the agent role. In this case, the first-pass parsing of the sentence, which would assign the role of agent to the grammatical subject, must be revised by a thematic re-analysis process that requires syntax and recruits additional cognitive resources.^2-4^

Damage to these processes has been reported repeatedly following left hemisphere damage. However, notwithstanding the impressive amount of research, the adoption of well-grounded theoretical frameworks and the use of sophisticated neuroimaging techniques, neurofunctional evidence is still controversial. This study addresses the neural correlates of thematic and morphosyntactic processes involved in the comprehension of reversible sentences.

### The role of temporal and parietal regions

Voxel-based lesion-symptom mapping (VLSM) studies have linked difficulties comprehending reversible sentences to left posterior damage. Most studies identified a parietal region including the angular and supra-marginal gyri^5-11^ and a posterior temporal region including the superior and middle temporal gyrus and the superior temporal sulcus^10,12-18^; some implicated also more anterior portions of the superior^6,7,19^ or of the middle temporal gyrus.^10^ The link between parieto-temporal structures and thematic role processing has been attributed to the role played by these regions in integrating semantic and syntactic knowledge^20-24^ or to their involvement in short-term memory processes.^12,25^ Evidence from neurotypical individuals largely converges with VLSM-based research. Left parieto-temporal hyper-activation was observed in functional MRI (fMRI) investigations,^26-29^ and repetitive Transcranial Magnetic Stimulation of the posterior third of the left intraparietal sulcus selectively affected comprehension of passive reversible sentences.^30-32^

Some VLSM investigations of thematic role reversals tackled the neurofunctional correlates of role assignment in greater detail, with variable outcomes. Factoring out phonological short-term memory did not modify significantly the temporo-parietal substrate retrieved in baseline analyses.^8,10^ Other studies focused on sentences with non-canonical word order (passives, object relatives, object clefts, etc.) or on the contrast between these and canonical stimuli (actives, subject relatives, subject clefts, etc.), with contradictory results. Most documented greater involvement of posterior temporal and parietal regions for non-canonical sentences.^5-7,14,16,27,33-37^ Others suggested that pre-frontal regions are involved, because non-canonicity and embedding activate area BA 44^38^ or because Broca’s area participates in the comprehension of complex non-canonical sentences, to the extent in which it involves working memory.^10,39^ Finally, yet other investigations reported no significant effects of word order.^5,8,40^ Notably, in all cases, conclusions were based on overall accuracy on mixed syntactic structures, ranging from simple passives (e.g. *The boy is kissed by the girl*) to very complex sentences (e.g. *Pete saw the girl who the man is pulling* or *The boy who the girl is pushed by is blue*), that do require non-canonical word order processing (hence, thematic re-analysis) but engage additional linguistic and cognitive processes, often to very different degrees. Stimulus heterogeneity may have contributed to unsystematic results, thus leaving the neural correlates of thematic re-analysis uncertain. In the present VLSM study, using only simple noun–verb–noun declaratives in the active and passive voice allowed investigating reliably the neural substrate involved in processing non-canonical word order.

### The role of pre-frontal regions

Poor performance on reversible sentences was reported often in individuals with Broca’s aphasia, whose lesions usually affect Broca’s area,^41-45^ and neuroimaging investigations in neurotypical participants documented activation in the left inferior frontal gyrus.^29,46-54^ In contrast, the results of VLSM research on the same issue are variable and controversial. Across studies on post-stroke aphasia, damage to Broca’s area: affected overall response accuracy on complex sentences but did not specifically correlate with thematic assignment errors^8^; disrupted the comprehension of words and sentences to a similar extent, suggesting an involvement in domain-general, rather than in syntax-specific processes^17^; correlated with performance in non-canonical (but not in canonical) sentence comprehension^7^; had only a complementary role in sentence comprehension^19^ and correlated with difficulty producing but not comprehending grammatical morphemes.^55^ Japanese participants with left pre-frontal gliomas fared poorly on a sentence–picture verification task, especially on passive sentences.^56,57^ Evidence from neurodegenerative conditions, albeit more frequently indicative of pre-frontal involvement, is also not straightforward. In primary progressive aphasia, frontal atrophy correlated with poor sentence comprehension,^58-60^ but in the context of damage to a larger network including postero-superior temporal regions and the intraparietal sulcus.^36^ In the non-fluent variant, a significant correlation between pre-frontal atrophy and poor sentence comprehension reported at baseline was no longer evident when short-term memory and single-word comprehension were factored out.^10^

Partly due to inconsistent evidence, the putative role of pre-frontal regions and especially of Broca’s area in sentence comprehension has received different interpretations.^61^ On some accounts, the area is involved in complex, language-specific syntactic operations, such as processing filler-gap dependencies,^44,62^ supporting structure-building or linearization operations,^38,63-68^ or integrating/unifying information into sentence context.^69^ On others, it contributes to sentence comprehension by allowing interactions between syntax and short-term memory.^51,68,70-78^ Yet, other accounts challenge the role of Broca’s area in basic linguistic mechanisms^39^ or posit an involvement limited to domain-general processes like the selection of semantic information among complex alternatives^79,80^ or the control of cognitive processes^24^ in computationally demanding circumstances such as the resolution of conflicting representations.^81^ Be this as it may, a large body of evidence suggests that Broca’s area is involved in sentence comprehension. Hence, failure to consistently find similar evidence in VLSM studies is puzzling.

The variability of the results could reflect limitations of published reports.^82^ Most VLSM investigations focused exclusively on thematic role assignment, which, albeit critical, is only one of the processes involved in sentence comprehension. Even in a simple sentence like *La ragazza è abbracciata dal ragazzo* [*La_f.sg._ ragazza_f.sg_ è_3rd.sg._ abbracciata_f.sg._ dal (da_prep_ + il_m.sg_) ragazzo_m.sg._;* The girl is hugged by the boy], morphosyntactic features (nominal and verbal inflections, subject/verb agreement, the *by*-phrase, etc) must be analysed to correctly assign the grammatical roles of subject and of agent complement, which are subsequently mapped onto the thematic roles of theme and agent, respectively. Consequently, morphosyntactic damage could interfere with grammatical role assignment and disrupt thematic analysis. Considering both morphosyntactic and thematic processes allows understanding the nature of thematic errors and their potential underlying heterogeneity. They might result indirectly from morphosyntactic damage in some cases and directly from thematic role assignment disruption in others, possibly due to lesions of distinct neurofunctional substrates.

The involvement of pre-frontal regions in morphosyntactic processes is suggested by the correlation of the inferior and middle frontal gyrus with morphosyntactic errors in aphasic speech^55,83,84^ and with morphosyntactic output manipulations during fMRI in neurotypical participants.^85-89^ However, the possibility of a correlation between pre-frontal regions and morphosyntactic processes in comprehension has never been investigated, possibly masking pre-frontal involvement and contributing to the uncertainty on its role.

The present VLSM study investigates morphosyntax and thematic role assignment in the same participants and in the comprehension of identical sentences. Considering both processes allows correlating lesions with damage to each process and evaluating pre-frontal involvement in morphosyntactic input processes.

## Materials and methods

### Participants and selection criteria

Seventy right-handed, native speakers of Italian with sub-acute or chronic aphasia following a first-ever left hemisphere stroke were considered. They were at least 18 years old and had received formal education for at least 8 years. None had a history of major psychiatric or medical comorbidities, including alcohol or drug abuse. All completed a computerized language battery.^90^ The experimental sample was selected solely on behavioural criteria, irrespective of clinical aphasia type and intra-hemispheric locus of lesion. It included individuals with poor performance on thematic or morphosyntactic foils but with normal scores on lexical-semantic foils, taken as a measure of difficulty with nouns and verbs in sentence context.

Behavioural inclusion criteria were met by 33 patients (17 female), who constituted the experimental group. All scored below norm on thematic foils. In 15 (10 female), morphosyntactic scores were pathological. None failed selectively on morphosyntactic foils. Seventeen patients were excluded because they scored normally on all foil types, and the remaining 20 because they scored below norm on all foil types (*n* = 11), on lexical and thematic (*n* = 6) or on lexical foils (*n* = 3). Patients were medically and cognitively stable throughout the language assessment procedures.

Ninety healthy volunteers (42 female) matched for age and education to the aphasic sample (Supplementary Table 1) completed the same sentence comprehension task.

The performance of neurotypical and aphasic participants is compared in Supplementary Table 2. Table 1 shows biographic and neuroradiological information on the patient group. Individual performance of aphasic participants on the experimental task is reported in Supplementary Table 3 and Supplementary Fig. 1.

**Table 1:** Demographic and lesion characteristics

Code	Sex	Age (years)	Education (years)	Aetiology	Test-onset (months)	Error	Scan	Voxel (mm^3)^	Volume (mm^3^)
1	F	22	13	HCVA	31	MS + TR	MR	1 × 1 × 7	100 891
2	F	76	8	HCVA	11	MS + TR	MR	1 × 1 × 7	23 483
3	F	40	11	Aneurysm	5	MS + TR	CT	1 × 1 × 1	233 725
4	F	53	18	ICVA	3	MS + TR	MR	1 × 1 × 1	45 009
5	F	34	13	ICVA	15	MS + TR	MR	1 × 1 × 1	23 643
6	F	73	13	ICVA	5	MS + TR	MR	1 × 1 × 1	54 886
7	F	30	15	ICVA	7	MS + TR	CT	1 × 1 × 1	180 680
8	M	67	17	PMLE	53	MS + TR	MR	1 × 1 × 1	136 440
9	M	53	17	ICVA	46	MS + TR	MR	1 × 1 × 1	122 851
10	M	44	17	ICVA	123	MS + TR	MR	1 × 1 × 1	143 849
11	M	24	13	ICVA	8	MS + TR	MR	1 × 1 × 1	145 581
12	F	73	13	ICVA	9	MS + TR	MR	1 × 1 × 1	79 667
13	F	65	8	ICVA	14	MS + TR	MR	1 × 1 × 1	19 445
14	M	57	10	ICVA	6	MS + TR	MR	1 × 1 × 1	133 867
15	F	69	10	Aneurysm	4	MS + TR	MR	1 × 1 × 1	89 147
16	M	53	18	HCVA	36	TR	MR	1 × 1 × 6	53 516
17	F	53	13	ICVA	1	TR	MR	1 × 1 × 1	22 279
18	F	47	16	HCVA	13	TR	MR	1 × 1 × 4	37 651
19	M	69	8	ICVA	32	TR	MR	1 × 1 × 1	158 009
20	M	57	13	ICVA	45	TR	MR	1 × 1 × 1	45 598
21	M	48	18	ICVA	13	TR	MR	1 × 1 × 6	207 685
22	M	39	13	ICVA	28	TR	MR	7 × 1 × 1	202 685
23	M	36	18	ICVA	30	TR	MR	7 × 1 × 1	84 026
24	F	60	8	ICVA	1	TR	MR	1 × 1 × 5	74 768
25	M	63	19	ICVA	9	TR	MR	1 × 1 × 1	45 407
26	F	45	18	ICVA	28	TR	MR	1 × 1 × 1	265 748
27	M	73	23	HCVA	34	TR	MR	1 × 1 × 1	82 288
28	F	58	13	ICVA	51	TR	CT	1 × 1 × 1	80 497
29	F	75	8	ICVA	34	TR	CT	1 × 1 × 1	5714
30	F	64	8	ICVA	4	TR	MR	1 × 1 × 1	39 712
31	M	78	8	ICVA	14	TR	MR	1 × 1 × 1	29 858
32	M	52	8	ICVA	6	TR	MR	1 × 1 × 1	66 178
33	M	61	8	ICVA	26	TR	MR	1 × 1 × 1	129 203

Code, patient identification reference; M, male; F, female; HCVA, haemorrhagic cerebrovascular accident; ICVA, ischaemic cerebrovascular accident; PMLE, progressive multi-focal leucoencephalitis; Test-onset, test time post-onset is expressed in months; TR, thematic role; MS, morphosyntactic; underlined, neuroimaging exams were analyzed based on TIFF source file; Volume, lesion volume.

Prior to participation, all participants gave informed consent, as required by the Helsinki Declaration.

### Behavioural assessment

#### Auditory sentence comprehension task

Sixty declarative (noun–verb–noun) reversible sentences were presented auditorily by computer, in random order. Twenty-nine items were in the active and 31 in the passive voice. Participants matched the stimulus sentence to one of two vertically arranged drawings. In each item, a drawing matched the auditory stimulus and the other was a foil. The position of targets and foils in the vertical array was counterbalanced; agents and themes appeared equally often in the right and left half of the drawings. Foils represented an equal number of times (*n* = 20) a thematic role reversal, a morphosyntactic alternative or a lexical-semantic alternative. Thematic and lexical-semantic foils were associated to 10 active and 10 passive stimuli; morphosyntactic foils were associated to 9 active and 11 passive stimuli. Morphological foils differed from the target in the number of the first or second noun. Lexical-semantic foils differed in the first noun, the second noun or the verb. The two morphosyntactic and the three lexical-semantic foils appeared a roughly equal number of times. Stimuli were divided in two sub-lists (*n* = 30 each), balanced for sentence voice and foil type (examples in Supplementary Fig. 2).

The performance of neurotypical subjects served to establish cut-off levels for thematic, morphosyntactic and lexical-semantic errors and to identify aphasic participants meeting the inclusion criteria. None of the controls made >2 errors on thematic foils or >1 error on morphosyntactic and lexical foils. Given the very low number of errors in neurotypical participants, the cut-off score was set at 3 SD below the controls’ mean. Descriptive statistics on the aphasic and the control sample are reported in Supplementary Table 2.

#### Verbal short-term memory tasks

Probe tasks allowed measuring phonological short-term memory independent of spoken output deficits. A word and a non-word task were administered, each consisting of 48 series (24 series of 4 bisyllabic stimuli and 24 series of 6 bisyllabic stimuli). In each series, stimuli were spaced by 1 s, and the probe item was presented 3 s after the last stimulus. The participant decided whether the probe had occurred in the series by providing a Yes/No response. The probe was present in half of the cases, an equal number of times in each within-series position. The cumulative error rate was considered when analysing the relationships between sentence comprehension and phonological short-term memory.

### Neuroimaging analysis

#### Data

For all participants, neuroradiological documentation of sufficient quality as to allow detailed lesion reconstruction was available (Table 1). Most structural scans were T1-weighted images from MRI exams (29 patients). CT images were available for the remaining four patients. For 26 patients, DICOM data were converted to NIfTI files. For the remaining seven patients, data were only available in TIFF format.

#### Processing

Lesions were reconstructed by manually drawing a region of interest (ROI) around the damaged area in FSL (version 6.0.5.1).^91^ The author (S.B.) involved in this step had no information on the patient’s functional profile until the entire lesion reconstruction process was completed. In patients (*n* = 7) for whom only TIFF images were available, the lesion was manually drawn in MNI space on the MNI152 1 × 1 × 1 mm template available in FSL. For all other participants, the ROI was drawn in the native space on either T1-weighted MR images or high-quality CT scan images. The ROI was delineated by identifying unequivocally damaged areas. Whenever doubts arose, the ROI was re-drawn based on consensus (S.B. and G.M.). Lesion ROIs were then mapped onto the MNI template by first re-sampling individual T1-weighted images to 1 × 1 × 1 mm using tri-linear interpolation in FSL and then mapping them to the MNI template using non-linear registration in FSL (FNIRT). Image normalization to the MNI152 space was carried out with ‘cost function masking’.^92,93^ The obtained warps were then applied to the ROIs using nearest neighbour interpolation. Lesion location in MNI space was based on the Harvard–Oxford cortical structural atlas.^94^ The mapping did not consider neuroanatomical structures overlapping with <10% of the cluster.

#### Statistical analysis

Voxel-based analyses (VLSM)^95^ were conducted in MRIcron using the Non-Parametric Mapping software^96^ (for information about method and software, see^97^). Only voxels damaged in at least 15% of the participants and with a minimum cluster size of 10 voxels (4000 permutations) were considered. All analyses were carried out on the entire 33-subject sample.

Analyses based on continuous behavioural measures (Brunner–Munzel test)^98^ were conducted to identify clusters correlated with severity of impairment on morphosyntactic and on thematic foils. A binary Liebermeister test^99^ was also conducted, contrasting damage/sparing of individual voxels with presence/absence of selective thematic role deficit or of morphosyntactic errors, to identify the neural correlates of pathological performance on morphosyntactic and on thematic foils, respectively.

All analyses were conducted on voxels with enough power to detect the lesion-symptom effect, based on the power map thresholded at *P* < 0.05 and corrected for multiple comparisons using the false discovery rate (FDR).

## Results

### Lesion volume and correlations with response accuracy

**Figure 1:**
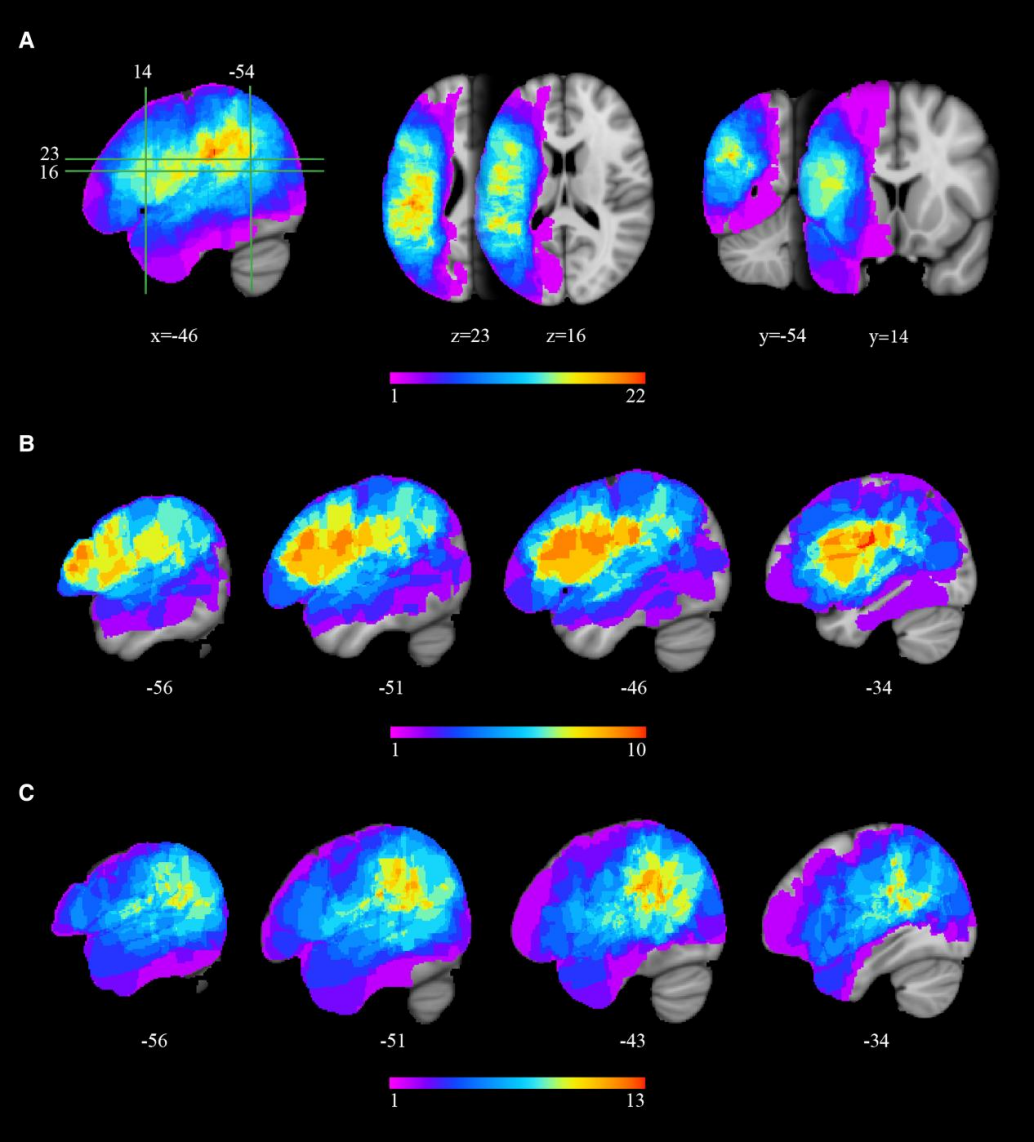
**Lesion overlap.** Lesion overlaps are shown on sagittal, axial and coronal slices, with MNI152 coordinates (in mm) below each slice. Colours indicate the number of patients contributing to the overlap, as indicated in the colour bars. (**A**) Lesions in the entire group (33 patients) covered a large portion of the left hemisphere, with pre-frontal and parieto-temporal maxima; the insula, the parietal operculum, the supra-marginal gyrus and a small portion of the angular gyrus were also damaged in all patients. (**B**) In patients with morphosyntactic errors (*n* = 15), lesions overlapped mostly in the inferior and middle frontal gyri, with extensive white matter involvement. Pre-central and post-central cortices were also affected. (**C**) In patients with selective role assignment errors (*n* = 18), lesions overlapped mostly in parieto-temporal regions, including the angular gyrus, and extending to the lateral occipital cortex.

Lesion overlap in the whole sample, in participants with morphosyntactic and thematic errors and in participants with selective thematic errors is shown in Fig. 1. The average lesion volume was 95 878.36 ± 67 691.28 mm^3^ in the participants with thematic processing difficulties (i.e. the whole sample, *n* = 33) and 102 210.9 ± 62 724.65 mm^3^ in the subgroup with co-occurring morphosyntactic impairment (*n* = 15 out of 33). Lesion size was distributed very similarly in this last subgroup and in 18 participants with selective thematic impairment (90 601.22 ± 72 939.69 mm^3^; Mann–Whitney U-test: *P* = 0.486).

Correlations between lesion volume and response accuracy were insignificant both in the overall sample (Spearman’s *rho* = −0.205; *P* = 0.253) and when the two patient subgroups were considered separately (15 participants with co-occurring error types, Spearman’s *rho* = 0.181; *P* = 0.52; 18 participants with selective thematic errors, Spearman’s *rho* = 0.317; *P* = 0.2).

Insignificant correlations between volume and overall accuracy contrasted with qualitatively different behavioural profiles. Thematic error rate was indistinguishable in the two subgroups (Student’s *t-*test: *t* = 0.569; *P* = 0.287; Hedges’ *g* = 0.194), but morphosyntactic errors were significantly more frequent in the subgroup with co-occurring error types (Student’s *t-*test: *t* = 7.659; *P* < 0.001; Hedges’ *g* = 2.789). Furthermore, morphosyntactic and thematic errors correlated significantly in this latter group (Spearman’s *rho* = 0.607, *P* = 0.008), but not in participants with selective thematic impairment (Spearman’s *rho* = 0.195, *P* = 0.219) (Supplementary Fig. 1).

Overall, data rule out that the contrasting performance profiles in the two subgroups are attributable to lesion volume.

### The neural correlates of morphosyntactic and thematic role assignment errors

**Figure 2:**
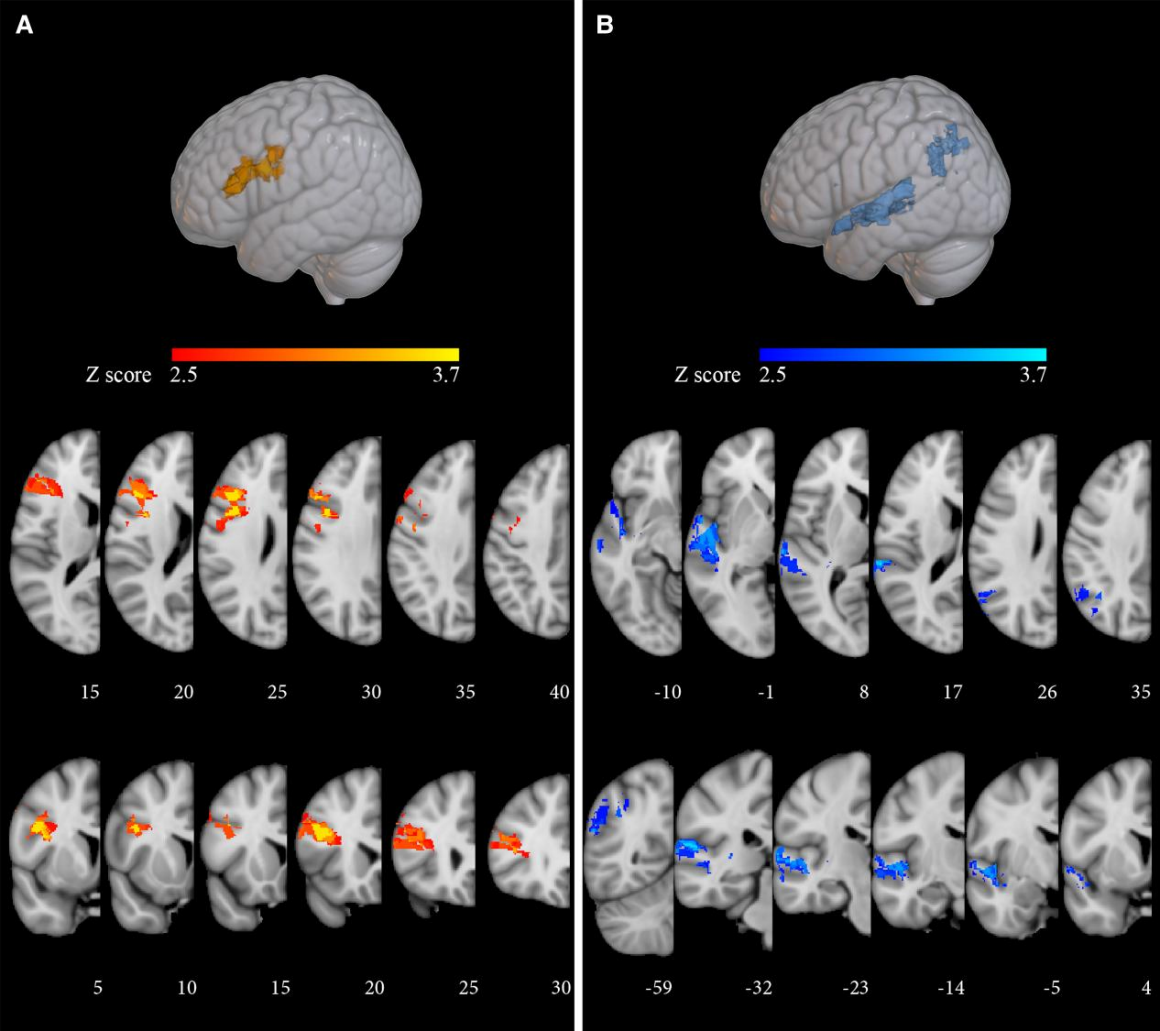
**VLSM Brunner–Munzel tests: correlation with severity of morphosyntactic processing and thematic role assignment deficits in the experimental sample (*n* = 33).** Voxels whose damage was significantly associated to poorer performance on morphosyntactic foils (**A**) or on thematic role assignment foils (**B**). The colour bars reflect the *Z*-scores of the significant clusters (*P* < 0.01, FDR corrected). Left hemispheric clusters are shown on 3D lateral views (top row) and on axial (middle row) and coronal slices (bottom row). MNI152 coordinates (in mm) along the *z-*axis (axial) and *y-*axis (coronal) are shown below each slice.

A first analysis (Brunner–Munzel tests) was aimed at identifying regions significantly related to the severity of thematic and morphosyntactic damage. It relied on binary voxel measures (damaged/spared voxel) and continuous behavioural measures (the number of morphosyntactic errors and of thematic reversals produced by each subject). Results yielded distinct neural correlations for morphosyntactic errors and thematic reversals. Poor performance on morphosyntactic foils correlated significantly with damage (VLSM *P* < 0.01; FDR corrected) to a large pre-frontal cluster (8831 mm^3^; centre of mass *x* = −44, *y* = 16 and *z* = 24 mm) including the pars opercularis and triangularis of the inferior frontal gyrus, the middle frontal gyrus and the pre-central gyrus (Fig. 2A; Table 2). In contrast, low accuracy on thematic foils correlated (VLSM *P* < 0.01; FDR corrected; 11 814 mm^3^; centre of mass *x* = −53, *y* = −30 and *z* = 11 mm) with posterior clusters including the angular gyrus, the posterior division of the superior temporal gyrus, the uppermost portion of the lateral occipital cortex, the planum temporale, the planum polare and a small portion of the posterior insular cortex (Fig. 2B; Table 2).

**Table 2:** VLSM analysis: correlation with severity of morphosyntactic processing and of thematic role assignment deficits

	Centre of mass							
Cluster	*x*	*y*	*z*	Volume (mm^3^)	Peak *Z*	Anatomical structures	%	Voxels
Morphosyntactic (*P* < 0.01, FDR corrected)								
1	−44	16	24	8831	3.66	Pre-central gyrus	24.07	2126
						Middle frontal gyrus	17.03	1504
						Inferior frontal gyrus, pars triangularis	15.92	1406
						Inferior frontal gyrus, pars opercularis	11.75	1038
Thematic role assignment (*P* < 0.01, FDR corrected)								
1	−55	−17	0	8340	3.72	Planum temporale	17.27	1440
						Superior temporal gyrus, posterior	15.38	1283
						Planum polare	12.84	1071
						Temporal pole	10.44	871
2	−41	−67	42	1883	3.66	Lateral occipital cortex, superior	92.25	1737
3	−53	−57	33	1520	3.3	Angular gyrus	77.96	1185
						Lateral occipital cortex, superior	16.84	256
4	−40	−6	−5	14	2.64	Insular cortex	100.00	14
5	−26	18	12	14	2.81	Insular cortex	78.57	14
6	−55	−72	24	13	2.82	Lateral occipital cortex, superior	100.00	13
7	−62	−59	16	11	2.53	Angular gyrus	100.00	11

%, percentage of the cluster (only structures covering at least 10% of the cluster are indicated); voxels, number of voxels (1 × 1 × 1 mm^3^) for each anatomical cluster.

**Figure 3:**
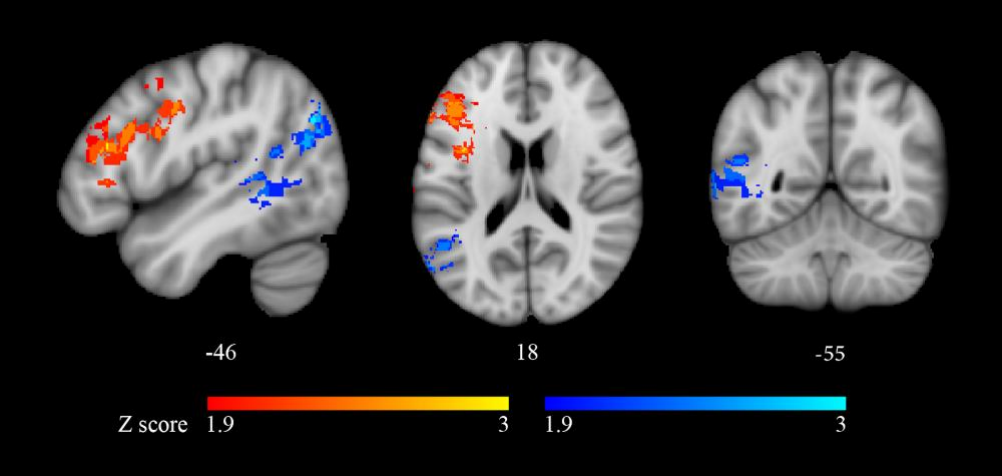
**VLSM Liebermeister tests.** Voxels whose damage is significantly associated with morphosyntactic difficulties (orange colour scale; *n* = 15), and voxels whose damage is significantly and selectively associated with thematic role difficulties (blue colour scale; *n* = 18). The colour bars reflect the *Z*-scores of the significant clusters (*P* < 0.05, FDR corrected). Clusters on the left hemisphere are shown, with MNI152 coordinates (in mm) shown below each slice (*x* on sagittal, *z* on axial, and *y* on coronal views).

An additional voxel-wise analysis (Liebermeister tests) was based on the binomial categorization of the patient sample as either making errors on morphosyntactic foils in addition to thematic foils (*n* = 15) or selectively responding below norm to thematic foils (*n* = 18) (Table 1). Binary measures were adopted both at the voxel level (damaged/spared voxel) and at the behavioural level (pathological/normal performance on morphosyntactic foils or only on thematic foils). Non-overlapping clusters were also retrieved in this case (Fig. 3; Supplementary Table 4). Damage to the pars triangularis and pars opercularis of the inferior frontal gyrus, to the middle frontal gyrus and to the pre-central gyrus distinguished individuals with versus without morphosyntactic impairment (*P* < 0.05; FDR corrected; 11 043 mm^3^; centre of mass: *x* = −46, *y* = 14 and *z* = 25 mm). In contrast, damage to the posterior division of the middle temporal gyrus, the superior division of the lateral occipital cortex, the planum temporale and the angular gyrus distinguished individuals who failed selectively on thematic foils from those who failed on both foil types (*P* < 0.05; FDR corrected; 6142 mm^3^; centre of mass: *x* = −50, *y* = −59 and *z* = 14 mm).

In light of the putative involvement of temporo-parietal regions in thematic role assignment, at face value the failure to find posterior damage in subjects with co-occurring morphosyntactic and thematic errors seems surprising. This issue is dealt with in the ‘Discussion’ section.

### Language processes and short-term memory in the comprehension of reversible sentences

The analyses reported so far link morphosyntactic and thematic processes to starkly distinguishable pre-frontal and parieto-temporal areas, respectively. However, the two clusters may have been retrieved not only because they are involved in strictly linguistic processes but also because they provide phonological short-term memory resources that maintain the auditory input string active during morphosyntactic and thematic analysis. To distinguish the role of language from that of short-term memory, results were analysed again in the 33 participants. Accuracy on morphosyntactic foils and on thematic foils was adjusted for short-term memory performance before VLSM analysis. A binary voxel measure (damaged/spared voxel) and continuous behavioural measures (errors on morphosyntactic foils and on thematic foils, covaried for errors on the memory probe task) were used.

**Figure 4:**
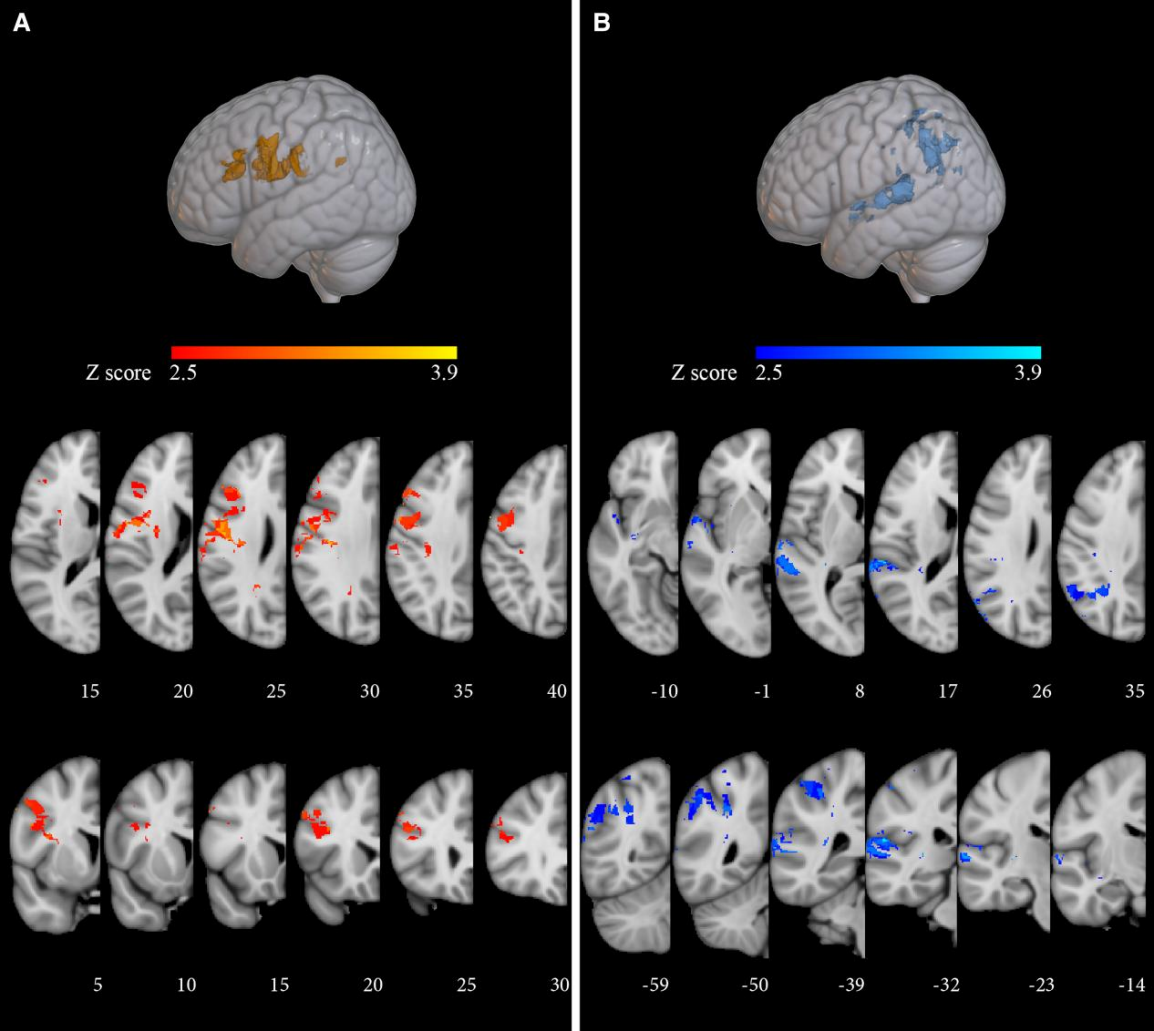
**VLSM: Brunner–Munzel tests with short-term memory as a covariate in the experimental sample (*n* = 33).** Voxels whose damage was significantly associated to poorer performance on morphosyntactic foils (**A**) or on thematic role assignment foils (**B**), with short-term memory as a covariate. The colour bars reflect the *Z*-scores of the significant clusters (*P* < 0.01, FDR corrected). Left hemispheric clusters are shown on 3D lateral views (top row) and on axial (middle row) and coronal slices (bottom row). MNI152 coordinates (in mm) along the *z-*axis (axial) and *y-*axis (coronal) are shown below each slice.

For morphosyntactic processes, the significant cluster (*P* < 0.01; FDR corrected; 8176 mm^3^; centre of mass: *x* = −46, *y* = −1 and *z* = 29 mm) included the pars opercularis and the pars triangularis of the inferior frontal gyrus, the middle frontal gyrus and the pre-central and post-central gyri (Fig. 4A; Table 3). For thematic processes, the significant cluster (*P* < 0.01; FDR corrected; 9526 mm^3^; centre of mass: *x* = −49, *y* = −44 and *z* = 29 mm) included the posterior division of the superior temporal gyrus towards the angular gyrus, the superior parietal lobule, the posterior division of the supra-marginal gyrus and the superior division of the lateral occipital cortex (Fig. 4B; Table 3). Both clusters were similar to, but more circumscribed than, those retrieved by the previous analyses (compare Figs 2A and 4A; Figs 2B and 4B; Tables 2 and 3). The correlates of morphosyntactic processes involved the same regions as the baseline analyses, whereas the cluster critical for thematic processes did not include the anterior and middle portions of the superior temporal gyrus. In conclusion, distinct neural substrates are critical for morphosyntactic and thematic processes, even when short-term memory is factored out.

**Table 3:** Voxel-based lesion mapping analysis: correlation with severity of morphosyntactic processing and of thematic role assignment deficits, covaried by short-term memory performance

	Centre of mass							
Cluster	*x*	*y*	*z*	Volume (mm^3^)	Peak *Z*	Anatomical structures	%	Voxels
Morphosyntactic processing with short-term memory as a covariate (*P* < 0.01, FDR corrected)								
1	−47	−5	29	6342	3.89	Pre-central gyrus	45.36	2877
						Post-central gyrus	15.67	994
2	−44	23	25	1659	3.51	Middle frontal gyrus	57.26	950
						Inferior frontal gyrus, pars triangularis	18.32	304
						Inferior frontal gyrus, pars opercularis	15.97	265
3	−49	−17	36	15	2.66	Post-central gyrus	100.00	15
Thematic role assignment with short-term memory as a covariate (*P* < 0.01, FDR corrected)								
1	−43	−57	37	4789	3.79	Angular gyrus	28.21	1351
						Lateral occipital cortex, superior	26.81	1284
						Supra-marginal gyrus, posterior	16.79	804
						Superior parietal lobule	11.32	542
2	−60	−30	9	3215	3.89	Superior temporal gyrus, posterior	35.80	1151
						Planum temporale	30.54	982
3	−41	−38	53	648	3.39	Superior parietal lobule	43.06	279
						Post-central gyrus	40.28	261
4	−48	−1	−6	186	3.17	Planum Polare	69.35	129
						Temporal pole	15.05	28
5	−33	−45	63	140	2.73	Superior parietal lobule	100.00	140
6	−52	−75	24	65	3.39	Lateral occipital cortex, superior	100.00	65
7	−33	−60	60	63	2.63	Superior parietal lobule	22.22	14
						Lateral occipital cortex, superior	77.78	49
8	−35	−10	−11	47	3.23	Insular cortex	19.15	9
9	−40	−30	24	47	2.95	Parietal operculum cortex	57.45	27
10	−31	−47	56	44	2.96	Superior parietal lobule	100.00	44
11	−40	−26	36	42	3.16	Post-central gyrus	38.10	16
12	−57	−31	23	41	2.79	Parietal operculum cortex	95.12	39
13	−28	−67	52	32	2.82	Lateral occipital cortex, superior	100.00	32
14	−52	−48	55	21	2.58	Supra-marginal gyrus, posterior	100.00	21
15	−36	−19	39	20	2.58	Post-central gyrus	80.00	16
16	−63	−55	25	12	2.54	Angular gyrus	100.00	12
17	−47	−74	36	10	2.63	Lateral occipital cortex, superior	50.00	10

%, percentage of the cluster (only structures covering at least 10% of the cluster are indicated); voxels, number of voxels (1 × 1 × 1 mm^3^) for each anatomical cluster.

### Non-canonical word order and the neural substrate of thematic re-analysis

Word order is a relevant dimension in comprehending reversible sentences. In actives, canonical word order facilitates interpretation. In passives, word order is non-canonical, and correctly assigning the agent role to the second noun requires thematic re-analysis, which entails a processing cost.^4^ As a first step towards identifying the neural substrate of thematic re-analysis, accuracy on passives was covaried for accuracy on actives in the entire sample. Thematic and morphosyntactic foils were analysed separately, as responses rely on different mechanisms in the two cases. For the same declarative passive sentence (e.g. *The woman is hugged by the girl*), identifying agent and theme in the presence of a thematic foil (a woman hugging a girl) requires considering the entire event, whereas focusing on the contrasting constituent suffices to decide between the correct picture and its morphosyntactic alternative (two women hugged by a girl; see examples in Supplementary Fig. 2). On these premises, non-canonical word order should affect performance on thematic foils, but not on morphosyntactic foils. Consistent with the prediction, error rate was significantly higher on passives than on actives in the presence of thematic alternatives (passives: 36.06 ± 18.87; actives: 28.18 ± 18.62; paired *t*-test: *t* = 1.831; *P* = 0.038; Hedges’*g* = 0.311), but not in the presence of morphosyntactic foils (passives: 10.12 ± 11.77; actives: 8.15 ± 10.79; *t* = 0.918; *P* = 0.183; Hedges’*g* = 0.156).

**Figure 5:**
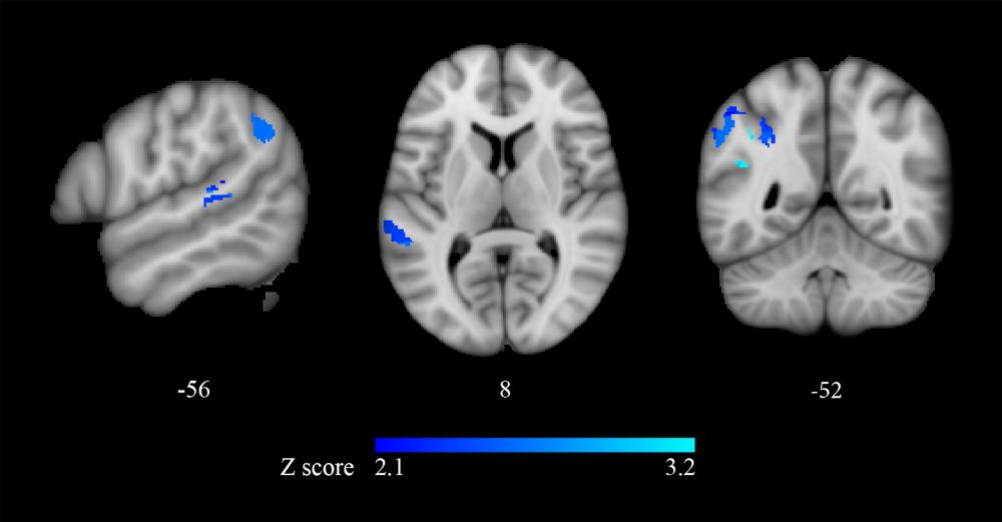
**VLSM Brunner–Munzel tests: correlation with thematic role assignment errors on passive sentences, with errors on active sentences as covariate in the experimental sample (*n* = 33).** Voxels whose damage is significantly associated to poorer performance on thematic role foils in passive sentences, with thematic errors in active sentences as a covariate. The results of the VLSM analysis on thematic errors with short-term memory as a covariate were used as an inclusive mask. The colour bar reflects the *Z*-scores of the significant clusters (*P* < 0.05, FDR corrected). Clusters on the left hemisphere are shown, with MNI152 coordinates (in mm) reported below each slice (*x* on sagittal, *z* on axial, and *y* on coronal views).

A follow-up VLSM-based analysis was then conducted to establish whether contrasting accuracy on passive versus active sentences yielded distinct lesion-symptom correlates. The analysis was restricted to the significant pre-frontal and posterior temporo-parietal clusters that survived covarying morphosyntactic and thematic errors for phonological short-term memory (Fig. 4; Table 3). A voxel-wise binary lesion measure (damaged/spared voxel) and a continuous behavioural measure (errors on morphosyntactic foils or on thematic foils in passive sentences, covaried by errors on the corresponding active sentences) were used. Significant clusters for thematic errors on passives were retrieved in the angular gyrus, the superior parietal lobule, the posterior division of the supra-marginal gyrus and of the superior temporal gyrus (*P* < 0.01; FDR corrected; 3327 mm^3^; centre of mass: *x* = −45, *y* = −48 and *z* = 35 mm; Fig. 5; Supplementary Table 5). In line with behavioural results, no significant clusters were retrieved for morphosyntactic errors.

## Discussion

The neurofunctional correlates of sentence comprehension were investigated in 33 individuals with post-stroke aphasia, via an auditory sentence–picture matching task including simple (noun–verb–noun) active and passive declarative sentences. Participants fared poorly on morphosyntactic or thematic contrasts but normally on lexical-semantic contrasts. Analyses focused on the neural correlates of sentence processing mechanisms. Results highlight a fronto-temporo-parietal network and help disentangle the role played by various components.

### Parieto-temporal regions and thematic role assignment

Our findings confirm the involvement of left parieto-temporal regions in thematic analysis.^5-9,11-19^ An initial evaluation of continuous behavioural data correlated thematic role assignment with the angular gyrus, the posterior division of the superior temporal gyrus, the planum polare, the planum temporale, the temporal pole and the superior division of the lateral occipital cortex (Fig. 2B). When accuracy on thematic foils was covaried for phonological short-term memory performance, error rate still correlated significantly with the same areas (Fig. 4B), except that the intermediate and anterior portions of the superior temporal gyrus were no longer retrieved. These latter areas have been implicated in short-term memory for auditory information,^100-103^ and their involvement could be task-specific, as in an auditory sentence–picture matching task early processing involves auditory cortices and acoustic radiations running deep to the insula.^104^ The temporal areas that survived covariance for short-term memory are implicated in the storage and retrieval of conceptual, lexical-semantic and lexical-grammatical information on verbs/actions^105^ and in processing verb argument structure.^106,107^ Parietal areas could be critical for the assignment of verb arguments to the correct thematic roles (this issue is addressed later).

### Pre-frontal regions and morphosyntactic processing

Neuroimaging studies link pre-frontal regions to sentence comprehension,^23,47,50,62,71,108^ but VLSM research supports this view unsystematically.^5-9,11-19,56,57^ In stroke patients, evidence is frequently negative^8,16,17,55^ or weak.^7,19^ In primary progressive aphasia, pre-frontal involvement is reported in the context of damage to a larger network including temporal and parietal areas^36,58-60^ but is no longer evident when short-term memory and word processing accuracy are factored out.^10^

Available VLSM studies are open to criticism, as they only considered thematic processes.^82^ Their results could weakly support the conclusion that pre-frontal areas are not critical for thematic role assignment but cannot rule out that they are otherwise involved in sentence interpretation. In the present project, morphosyntactic and thematic processes were assessed in all participants, in structurally identical sentences. Results clearly link morphosyntactic processing to the pars opercularis and the pars triangularis of the inferior frontal gyrus and to the lower portion of the middle frontal gyrus, with extensive white matter involvement (Fig. 2A). The same structures were retrieved in a binary analysis that categorized the 15 participants who fared poorly on morphosyntactic foils as distinct from the 18 who made only role reversals (Fig. 3).

Our results support a direct involvement of pre-frontal structures in morphosyntactic processes, rather than assigning them an ancillary role in sentence comprehension. The cluster retrieved in the baseline analysis (Fig. 2A) may include areas involved in short-term memory processes.^75,76,109^ However, after factoring out phonological short-term memory, pre-frontal damage still correlated significantly with severity of morphosyntactic impairment, in smaller clusters but in the same areas (Fig. 4A). In addition, if the only role of pre-frontal areas were to provide memory resources for sentence comprehension, accuracy on morphosyntactic foils should be lower on passives, which engage short-term memory more than actives. That morphosyntactic errors on simple declaratives were unaffected by sentence voice and occurred in basic contrasts, suggests that the pre-frontal cluster in Fig. 4A plays a direct role in local morphosyntactic processes.

This claim does not align with previous suggestions that pre-frontal regions are critical for complex syntactic operations.^38,44,62-67,69^ The apparent contrast could arise from the choice of different stimuli across investigations. We administered simple declarative sentences that only require local morphosyntactic analysis, whereas studies advocating a pre-frontal role in complex syntactic processes included stimuli containing long-distance dependencies.^110^ Results could be accounted for by assuming that morphosyntactic analysis is the only language skill of pre-frontal regions and that memory resources are recruited ‘as needed’, possibly in the same areas, when complex morphosyntactic dependencies are processed. As an alternative, pre-frontal areas could possess additional language skills, which are recruited when processing complex syntactic structures.^111^

### Pre-frontal and temporo-parietal areas: the heterogeneity of thematic role errors

Pathological numbers of role reversals were observed in all patients, but in the context of distinct performance profiles. They were the sole error type in 18 and co-occurred with morphosyntactic errors in 15. A binary analysis (Liebermeister test) showed distinct neurofunctional correlates for the two profiles. Selective thematic errors correlated with the middle temporal gyrus, the supra-marginal and the angular gyri, whereas role reversals co-occurring with morphosyntactic errors were linked to the inferior and middle frontal gyri and the underlying white matter (Fig. 3). Different anatomical and behavioural contexts document the heterogeneity of thematic errors and offer an opportunity to disentangle the neurofunctional mechanisms underlying the comprehension of morphosyntactic and thematic sentence features.

### Co-occurring morphosyntactic and thematic errors

In the overall sample, morphosyntactic errors correlated with pre-frontal damage. In the 15 participants who fared poorly on morphosyntactic foils, scores on thematic foils were always pathological (Table 1). Such error co-occurrence cannot be attributed to lesion volume, as already discussed. Alternatively, one could assume that morphosyntactic and thematic processes are functionally independent but overlap in pre-frontal regions, so that damage to this area affects both. This is reasonable but unlikely, given the number of VLSM studies that failed to correlate pre-frontal damage with thematic errors.^5,6,8,9,11-18^ A more likely hypothesis is that left pre-frontal areas are critical for morphosyntactic processes and that role reversals are inevitable when these processes are damaged. On this view, inferior and middle frontal damage would disrupt morphosyntactic analysis, thus interfering with the assignment of grammatical roles (subject, direct object, agent complement, etc.). Under-specified grammatical information reaching the temporal and parietal regions tasked with thematic analysis would ultimately yield role reversals.

The view that pre-frontal areas are involved directly in morphosyntactic processing but systematically influence thematic processing predicts that one should not find patients with selective morphosyntactic deficit in comprehension. This holds true in our sample, as pathological numbers of role reversals were invariably observed in the 15 participants who fared poorly on morphosyntactic foils. Furthermore and strengthening the possibility that thematic errors associated with pre-frontal damage are secondary to the inability to process local morphosyntax, rather than from a direct role of these structures in thematic analysis, in these participants morphosyntactic and thematic errors were significantly correlated (Spearman’s *rho* = 0.607; *P* = 0.008).

Role reversals associated with pre-frontal lesions occurred equally often on passive and active stimuli (paired *t*-test: *t* = 0.13; *P* = 0.449; Hedges’*g* = 0.032). This result is apparently counterintuitive, as interpreting passives requires processing both passive morphosyntax and non-canonical word order. However, since Italian passive sentences contain more morphosyntactic cues than actives (Supplementary Fig. 2 for examples), it is possible that even in the presence of morphosyntactic damage enough agreement cues remain available for sentence interpretation if thematic processes *per se* are intact. The assumption that pre-frontal areas are critical for morphosyntactic analysis and that morphosyntactic deficits result in thematic role reversals accommodates the failure to find temporo-parietal damage in patients with pre-frontal damage and co-occurring error types.

Before discussing the correlates of thematic processes, three issues should be briefly considered.

Faced with results that unequivocally point to pre-frontal involvement in morphosyntactic comprehension, one must account for why previous studies failed to systematically provide similar evidence. The most obvious reason is that they did not investigate morphosyntax.^82^ In addition, they measured role assignment skills by collapsing performance across a variety of sentences, including complex stimuli that need thematic (re)analysis but engage additional linguistic, cognitive and memory resources. Without explicitly probing morphosyntactic analysis and given the heavy burden on multiple subsidiary processes, pre-frontal involvement may have been masked.

Language choice may have also played a role. Sentence comprehension is influenced by several cues, whose relative strength varies across languages. Cross-linguistic studies showed that word order is a stronger cue in English and German than in Italian, whereas agreement cues are stronger in morphologically richer Italian and German than in English.^112,113^ Since most lesion-symptom investigations focused on thematic processes and were carried out in English,^82^ the paucity of studies showing pre-frontal involvement could result from the fact that in English, thematic analysis (carried out in temporo-parietal areas) plays a greater role in comprehension than morphosyntactic operations (carried out in pre-frontal structures). In indirect support of this account, the few VLSM studies reporting pre-frontal involvement were conducted in morphologically complex languages like Icelandic,^7^ Japanese^56,57^ and Korean.^19^ Also these investigations considered only thematic contrasts, but pre-frontal clusters could have been retrieved because unassessed morphosyntactic damage may have caused role reversals.

When considered in combination with studies on production in aphasic^55,82,84,114^ and neurotypical participants,^85-89^ comprehension data suggest that the inferior and middle frontal gyri might be critical for morphosyntactic processing in both modalities. The literature on aphasia is puzzling with this regard. Selective morphosyntactic errors were documented repeatedly in speech,^115-119^ but never in comprehension. In our study, they always coexisted with thematic errors. The hypothesis that pre-frontal areas are directly involved in morphosyntactic but not in thematic analysis accommodates this apparent contrast. Thematic roles are processed before morphosyntax in production, but after morphosyntax in comprehension. Hence, in production selective morphosyntactic damage may cause ungrammatical speech but spare thematic role assignment, whereas in comprehension morphosyntactic deficits will cause errors of both types.

### Selective deficit of thematic analysis

The account offered for the thematic errors co-occurring with morphosyntactic errors in the 15 patients with pre-frontal damage is untenable for the role reversals observed in the 18 participants with exclusively thematic errors. In contrast with the previous subgroup, they presented damage (Fig. 3) to temporo-parietal structures representing lexical-semantic information on verbs and actions^105^ and verb argument structure.^106,107^ The neural distinction between the two subgroups corresponds to a behavioural distinction as, contrary to voice-independent role assignment accuracy in morphosyntactic damage, selective thematic difficulty affected passives significantly more than actives, suggesting that performance in this case is influenced by difficulty in thematic re-analysis.

Since all stimuli in this project were simple declaratives, analysing the effect of sentence voice on response accuracy to thematic foils allowed investigating the neurofunctional correlates of thematic re-analysis on a homogeneous set of sentences. After factoring out performance on actives, accuracy on passives correlated with a parietal cluster including the angular and supra-marginal gyri and the superior parietal lobule (Fig. 5). Interestingly, these regions were implicated in the comprehension of passive sentences in neuromodulation^30-32^ and neuroimaging investigations.^22,35,120,121^ Our results are apparently at odds with those of fMRI studies, in which passive sentences also activated the inferior frontal gyrus.^22,35,56,120,121^ However, pre-frontal activation in these studies could be attributed to the (necessary but unexplored) morphosyntactic analysis of the target sentence, rather than to thematic re-analysis.

## Conclusion

Comprehending reversible sentences requires morphosyntactic processes, thematic processes and memory resources, represented in a large fronto-temporo-parietal network. Evidence from aphasia converges with neuroimaging studies that repeatedly documented simultaneous activation in the same network.^10,36,58-60,122-126^ Our offline behavioural and neuroimaging data cannot capture the directionality of the interactions, nor the network activity in real time (for neurophysiological data, see^111^). However, they demonstrate that areas in the sentence comprehension network play direct but distinct roles in sentence processing, perhaps via the coexistence of language-specific and domain-general processes in each (as suggested in this and other studies^8,10^ by the persistence of clusters in the same areas retrieved at baseline after partialling out phonological short-term memory).

A pre-frontal region including the inferior and middle frontal gyri and extending into subcortical white matter strongly correlates with morphosyntactic processes. The correlation persists after covarying for phonological short-term memory and is insensitive to sentence voice, suggesting that these areas process local features (e.g. determiner–noun and noun–verb agreement, the *by*-phrase). Damage to this area disrupts the assignment of grammatical roles, indirectly leading to thematic role errors. Role reversals following pre-frontal lesions could result from cortical damage or from pre-frontal and temporo-parietal areas being disconnected. It remains to be seen whether pre-frontal structures are involved exclusively in local morphosyntactic processing. Our results apply to simple declaratives, which contain only short-distance dependencies, but pre-frontal areas could intervene on more complex syntactic structures by recruiting short-term memory resources or by performing additional language operations.

Temporal and parietal areas are more directly linked to thematic role assignment. A cluster was retrieved in the posterior division of the superior temporal gyrus and the lateral occipital cortex, correlated with the representation of action knowledge and verb argument structure in previous neuroimaging studies. Another cluster including the supra-marginal and angular gyri survived corrections for phonological short-term memory and for accuracy on active sentences. This area might integrate information from pre-frontal and posterior temporal areas and implement thematic (re)analysis, critical for processing non-canonical word order.

Thematic errors in sentence comprehension are heterogeneous. In pre-frontal lesions, they are a consequence of morphosyntactic impairment. In parieto-temporal lesions, they result from failure to retrieve verb argument structure or from damage to processes critical for thematic role assignment and especially to thematic re-analysis. Such heterogeneity must be considered when investigating the neurofunctional correlates of thematic mapping.

It is not suggested here that each cluster intervenes only in the linguistic process with which it correlates in VLSM analyses, nor that each process only recruits the cluster to which it is linked. Sentence comprehension involves a large network of left hemisphere areas, each playing a prominent (but, not necessarily exclusive) role in specific sentence comprehension stages and interacting with other regions of the network.

## Supplementary Material

fcaf093_Supplementary_Data

## Data Availability

The conditions of our ethical approval do not permit public archiving of anonymized patient data. All data necessary for reproducing the results in this article can be requested from the first author.
