# Supplementary material for: Distinct neural correlates of morphosyntactic and thematic comprehension processes in aphasia
Source: Brain Commun. 2025 Mar 24;7(2):fcaf093. doi: 10.1093/braincomms/fcaf093 (PMC11930358; doi:10.1093/braincomms/fcaf093)
Supplement: fcaf093_Supplementary_Data [file fcaf093_supplementary_data.pdf]

Supplementary Table 1 Main epidemiological features of the experimental samples

	Age	Education
**Overall patient sample (N=33)**	54.9±15.3	13.2±4.2
**Patients with morphosyntactic and thematic errors (N=15)**	52.00±18.65	13.07±3.26
**Patients with selective thematic errors (N=18)**	57.28±11.94	13.22±4.97
**Healthy controls (N=90)**	58.2±12.8	12.0±4.0

Age and education were contrasted (Student's *t* test) in participants with aphasia and in healthy controls. No significant differences emerged.

a) Overall patient sample (n=33) vs healthy controls (n=90):
age: t = -1.19; p = 0.236; education: t = 1.16; p = 0.25;

b) Patients with morphosyntactic and thematic errors (N=15) vs healthy controls (n=90):
age: t = -1.229; p = 0.237; education: t = 0.798; p = 0.427;

c) Patients with selective thematic errors (N=18) vs healthy controls (n=90):
age: t = -0.266; p = 0.791; education: t = 0.952; p = 0.343;

d) Patients with morphosyntactic and thematic errors (N=15) vs Patients with selective thematic errors (N=18)
age: t = -0.946; p = 0.354; education: t = -0.108; p = 0.915.

Supplementary Table 2 Error percentage in the overall patient sample, in participants with co-occurring morphosyntactic and thematic errors, in participants with selective thematic errors is reported and contrasted with error percentage in neurotypical controls.
NB. Comparisons between participants with co-occurring morphosyntactic and thematic errors and with exclusively thematic errors are reported in the main text.

	Sentence comprehension		Memory
	Thematic roles	Morphosyntax	
**Overall patient sample (N=33)**	32.1 ±14.1	9.2 ±9.4	17.7 ±9.5
**Patients with morphosyntactic and thematic errors (N=15)**	33.7 ±16.4	17.7 ±7.3	18.3 ±7.8
**Patients with selective thematic errors (N=18)**	30.8 ±12.2	2.2 ±3.1	17.2 ±11
**Healthy controls (N=90)**	1.9 ±2.9	0.3 ±1.3	Not administ

Response accuracy was contrasted (Student's *t* test) in participants with aphasia and in healthy controls.

a) Overall patient sample (N=30) vs healthy controls (N=90).
Compared to neurotypical participants, patients made significantly more thematic errors (t=12.235; p<0.001), with a very large effect size (g=3.928), and morphosyntactic errors (t=5.4; p<.001), with a large effect size (g=1.779).

b) Patients with morphosyntactic and thematic errors (N=15) vs healthy controls (N=90).
Compared with neurotypical participants, patients made significantly more thematic errors (t=7.478; p<0.001), with a very large effect size (g=4.771), and morphosyntactic errors (t=9.19; p<0.001), with a very large effect size (g=5.876).

c) Patients with selective thematic errors (N=18) vs healthy controls (N=90).
Compared with neurotypical participants, patients made significantly more thematic errors (t=10.046; p<0.001), with a very large effect size (g=5.197), and morphosyntactic errors (t= 2.561; p<0.02), with a large effect size (g=1.113).

Supplementary Table 3. Error percentage in the sentence comprehension task (total N of stimuli = 60) and in the memory probe task (total N of stimuli = 96) in each participant with aphasia. Some participants were available for a very short time and completed shortened versions of the tasks, as indicated by footnotes.

	Thematic roles			Morphology			Semantics			Memory
Code	Active	Passive	Total	Active	Passive	Total	Active	Passive	Total	
1	40	40	40	22	18	20	0	10	5	15
2[a]	60	20	40	20	40	30	0	0	0	33[b]
3	30	40	35	22	18	20	10	0	5	23
4	20	40	30	11	18	15	0	10	5	19
5	40	40	40	22	27	25	0	0	0	24
6	10	20	15	0	18	10	0	10	5	14
7	40	70	55	11	9	10	0	0	0	14
8[a]	80	60	70	20	40	30	0	0	0	21[c]
9	20	20	20	20	0	10	17	0	5	4[c]
10	10	40	25	11	18	15	0	0	0	17[b]
11	10	30	20	22	18	20	10	0	5	18
12	20	10	15	0	27	15	0	0	0	17
13	30	20	25	0	18	10	10	0	5	14
14	60	50	55	44	9	25	0	10	5	33[b]
15	30	10	20	11	9	10	0	0	0	11
16[a]	40	0	20	0	0	0	0	0	0	13[b]
17	10	40	25	11	0	5	0	10	5	18
18[a]	0	60	30	0	0	0	0	0	0	25[b]
19	10	30	20	11	0	5	10	0	5	13
20	30	20	25	0	0	0	0	0	0	20
21	20	60	40	11	0	5	0	0	0	13
22[a]	60	60	60	0	0	0	17	0	10	27[b]
23	50	50	50	0	0	0	0	0	0	17[b]
24	10	40	25	0	0	0	0	10	5	16
25[a]	40	20	30	0	20	10	0	0	0	23[b]
26	30	30	30	0	9	5	0	10	5	12[c]
27[a]	20	60	40	0	0	0	0	0	0	17[b]
28	20	10	15	0	0	0	0	0	0	8
29	30	30	30	0	0	0	0	10	5	6
30	20	10	15	0	0	0	10	0	5	24
31	20	60	40	0	9	5	10	0	5	51
32	0	40	20	0	0	0	0	0	0	8
33	20	60	40	0	9	5	0	0	0	0

[a] Shortened version of the sentence comprehension task (N = 30 stimuli). These participants responded to 5 active and 5 passive stimuli with morphosyntactic foils.

[b] Shortened version of the memory probe task (N = 48 stimuli).

[c] Shortened version of the memory probe task (N = 24 stimuli).

Supplementary Table 4 Voxel-based lesion-symptom mapping analysis (Liebermeister tests): correlates of pathological performance on morphosyntactic foils and on thematic foils

Presence/absence of morphosyntactic difficulties (*P* < 0.05, FDR-corrected)

	Centre of mass							
**Cluster**	**x**	**y**	**z**	**Volume**	**Peak Z**	**Anatomical structures**	**%**	**voxels**
1	-46	14	26	10532	3.03	Precentral Gyrus	33.94%	3575
						Middle Frontal Gyrus	23.03%	2425
						Inferior Frontal Gyrus, pars triangularis	17.19%	1810
						Inferior Frontal Gyrus, pars opercularis	17.19%	1763
2	-32	8	1	105	2.57	Insular Cortex	100.00%	105
3	-46	30	3	88	2.57	Inferior Frontal Gyrus, pars triangularis	100.00%	88
4	-65	-19	37	73	2.93	Postcentral Gyrus	98.63%	72
5	-67	-22	22	48	2.33	Postcentral Gyrus	58.33%	28
						Supramarginal Gyrus, anterior division	41.67%	20
6	-27	3	43	32	2.58	Middle Frontal Gyrus	93.75%	30
7	-39	-17	31	26	2.19	Precentral Gyrus	76.92%	20
						Postcentral Gyrus	19.23%	5
8	-51	5	22	20	2.17	Precentral Gyrus	100.00%	20
9	-55	8	7	17	2.17	Precentral Gyrus	88.24%	15
						Inferior Frontal Gyrus, pars opercularis	11.76%	2
10	-48	-6	53	17	1.97	Precentral Gyrus	100.00%	17
11	-31	33	-2	16	1.97	Frontal Pole	50.00%	8
						Middle Frontal Gyrus	31.25%	5
						Inferior Frontal Gyrus, pars triangularis	18.75%	3
12	-28	16	11	13	2.22	Insular Cortex	100.00%	13

Presence/absence of selective thematic role difficulties (*P* < 0.05, FDR-corrected)

	Centre of mass							
**Cluster**	**x**	**y**	**z**	**Volume**	**Peak Z**	**Anatomical structures**	**%**	**voxels**
1	-51	-59	13	5799	3.01	Middle Temporal Gyrus, temporooccipital	36.99%	2145
						Lateral Occipital Cortex, superior	34.63%	2008
2	-30	-67	54	107	2.43	Lateral Occipital Cortex, superior	100.00%	107
3	-66	-45	-2	39	2.43	Middle Temporal Gyrus, temporooccipital	64.10%	25
						Middle Temporal Gyrus, posterior	35.90%	14
4	-38	-69	47	26	2.09	Lateral Occipital Cortex, superior	100.00%	26
5	-37	-69	23	19	2.47	Angular Gyrus	52.63%	10
						Lateral Occipital Cortex, superior	47.37%	9
6	-39	-42	15	15	1.93	Planum Temporale	100.00%	15
7	-52	-68	42	14	2.13	Lateral Occipital Cortex, superior	100.00%	14
8	-53	-36	-5	13	2.09	Middle Temporal Gyrus, posterior	50.00%	13
9	-37	-78	40	13	2.09	Lateral Occipital Cortex, superior	50.00%	13

Volume is expressed in mm^3; % = percentage of the cluster (only structures covering at least 10% of the cluster are indicated); voxels = number of voxels (1x1x1 mm^3) for each anatomical cluster.

Supplementary Table 5 Voxel-based lesion-symptom mapping analysis: correlates of thematic error rate on passive sentences with errors on active sentences as a covariate, while controlling for short-term memory performance (*P* < 0.05, FDR-corrected)

	Centre of mass							
**Cluster**	**x**	**y**	**z**	**Volume**	**Peak Z**	**Anatomical structures**	**%**	**voxels**
1	-41	-52	41	2314	3.17	Angular Gyrus	33.97%	786
						Supramarginal Gyrus, posterior	29.73%	688
						Superior Parietal Lobule	28.65%	663
2	-60	-33	9	579	2.84	Superior Temporal Gyrus, posterior	71.16%	412
						PlanumTemporale	28.32%	164
3	-35	-38	50	229	2.7	Postcentral Gyrus	56.77%	130
						Superior Parieta Lobule	43.23%	99
4	-45	-53	24	68	3.17	Angular Gyrus	100.00%	68
5	-60	-38	16	64	2.13	Planum Temporale	90.62%	58
6	-61	-29	15	27	2.28	Planum Temporale	88.89%	24
						Parietal Operculum Cortex	11.11%	3
7	-47	-56	33	25	2.22	Angular Gyrus	100.00%	25
8	-52	-48	55	21	2.25	Supramarginal Gyrus, posterior	100.00%	21

Volume is expressed in mm^3; % = percentage of the cluster (only structures covering at least 10% of the cluster are indicated); voxels = number of voxels (1x1x1 mm^3) for each anatomical cluster.

Supplementary Figure 1 Shows the percentage of morphosyntactic and of thematic errors produced by each participant in the sentence comprehension test. Participants with co-occurring morphosyntactic and thematic errors are represented by blue circles, participants with selective thematic errors by purple triangles. For display purposes, *ex-aequo* data points were slightly jittered to distinguish participants with identical performance.

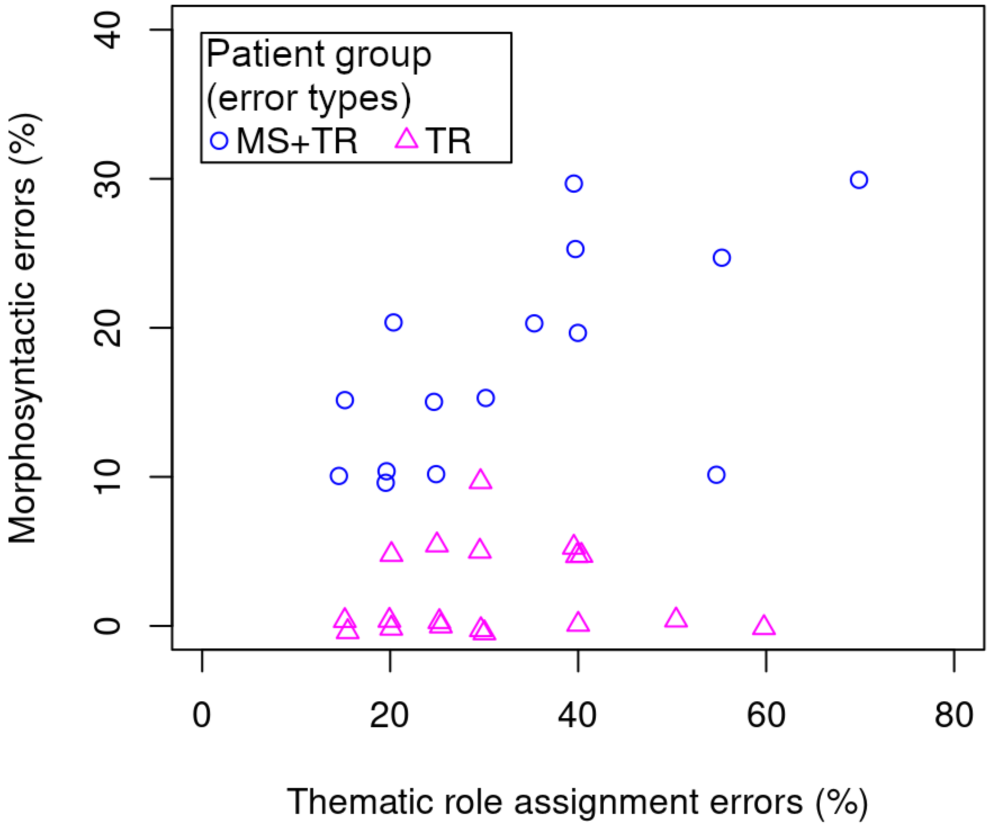

Supplementary Figure 2 Examples of items of the Auditory Sentence Comprehension Task

Thematic Foils

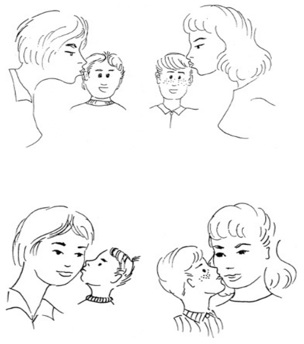

Le mamme baciano i bambini
The mothers kiss the boys

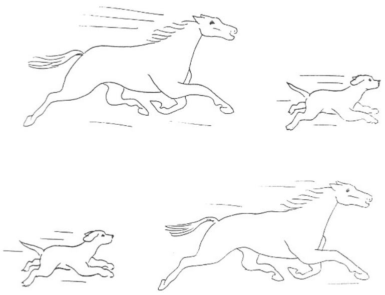

Il cavallo è inseguito dal cane
The horse is chased by the dog

Morphosyntactic Foils

(Morphosyntactic cues are underlined in each stimulus

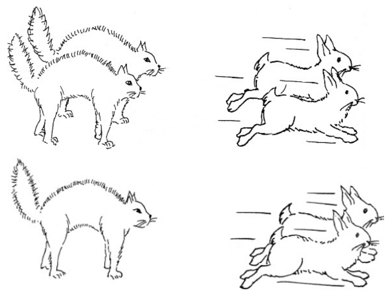

I gatti spaventano i conigli
The cats scare the rabbits

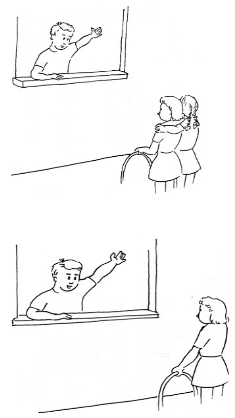

Il bambino saluta le bambine
The boy greets the girls

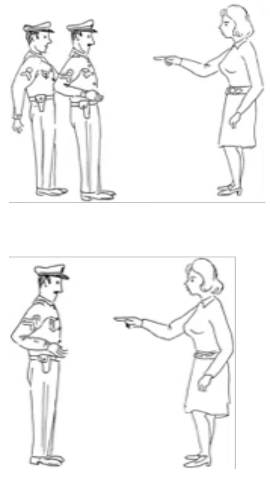

Il poliziotto è indicato dalla donna
The policeman is indicated by the woman

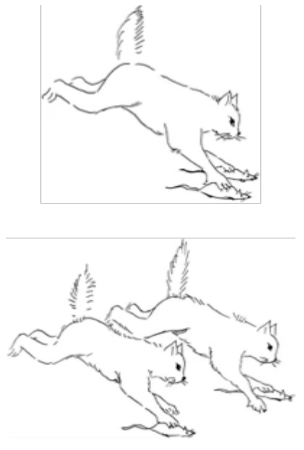

I topi sono assaliti dal gatto
The mice are assaulted by the cat

Lexical-Semantic Foils

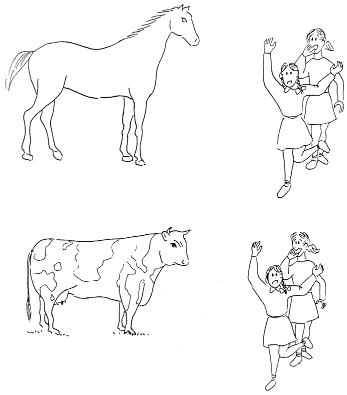

Il cavallo spaventa le bambine
The horse scares the little girls

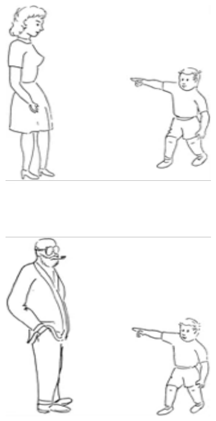

Il bambino indica il nonno
The boy indicates the granfather

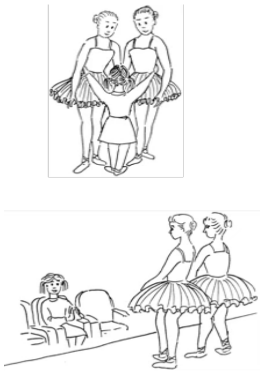

La bambina abbraccia le ballerine
The girl hugs the ballerinas

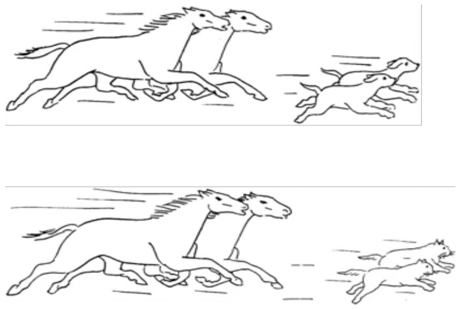

I gatti sono inseguiti dai cavalli
The cats are chased by the horses

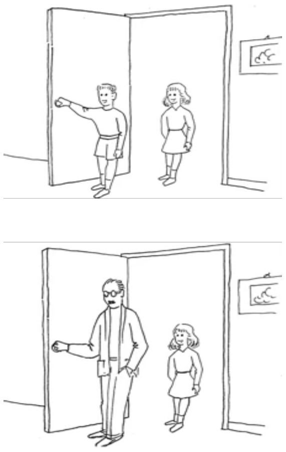

La bambina è preceduta dall’uomo
The girl is preceded by the man

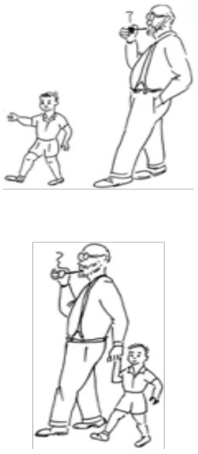

Il bambino è seguito dal nonno
The boy is followed by the old man
